# Optimization extraction of Allium mongolicum Regel polysaccharide and alleviation of intestinal injury via inhibition of the PERK/ATF4/CHOP signaling pathway

**DOI:** 10.3389/fvets.2026.1773978

**Published:** 2026-03-26

**Authors:** Xiaoyu Niu, Guorong Nan, Yankai Zheng, Yu Chen, Huinan Jiang, Jing Zhang, Lu Chen, Yuanyuan Xing, Dabiao Li

**Affiliations:** 1 College of Animal Science, Inner Mongolia Agricultural University, Hohhot, China; 2 Key Laboratory of Animal Nutrition and Feed Science at Universities of Inner Mongolia Autonomous Region, Hohhot, China; 3 Intellectual Property Protection Center of Inner Mongolia Autonomous Region, Hohhot, China; 4 Animal Husbandry and Veterinary Department, Shanxi Animal Husbandry and Veterinary School, Taiyuan, China

**Keywords:** Allium mongolicum Regel polysaccharides, diquat, endoplasmic reticulum stress, immunity, mitochondrial autophagy

## Abstract

**Introduction:**

This study aimed to optimize the extraction conditions of *Allium mongolicum Regel polysaccharides* (*AMRP*) and investigate how *AMRP* alleviates diquat (*DQ*)-induced intestinal injury by modulating endoplasmic reticulum stress (ERS).

**Methods:**

This experiment consisted of two parts. 1)Optimizing the optimal extraction conditions for AMRP using single-factor experiments combined with response surface methodology (RSM).2) *In vivo* experiment: A total of 36 specific pathogen-free (SPF) mice were selected and randomly divided into 3 treatment groups, namely the control group (CON), diquat group (DQ), and AMRP + diquat challenge group (AMRP+DQ). The experiment included a 7-day pre-feeding period and a 21-day formal trial period, during which the mice had free access to drinking water and feed. Mice in the AMRP+DQ group were gavaged with 0.2 ml of AMRP solution at a concentration of 80 mg/ml, while mice in the CON and DQ groups were gavaged with the same volume of normal saline. One day before the end of the experiment, mice in the DQ and AMRP+DQ groups were subcutaneously injected with DQ solution at a concentration of 10 mg/kg body weight (BW). After a 12-h fast, the mice were slaughtered for sample collection.

**Results:**

Supplementation with *AMRP* significantly increased thymus and spleen indices, improved intestinal morphology, markedly reduced serumlevels of pro-inflammatory cytokines (*P* < 0.05), elevated serum anti-inflammatory factors (*P* < 0.05), and decreased pro-inflammatory cytokine levels in intestinal tissues of DQ-challenged mice.

**Conclusion:**

*AMRP* suppressed the expression of key factors in the ERS signaling pathway (PERK/ATF4/CHOP). In conclusion, the optimal extraction conditions for *AMRP* were 25% papain, 4.5 h hydrolysis, and 63 °C. *AMRP* effectively reduced inflammation, alleviated *DQ*-induced intestinal injury via the PERK/ATF4/CHOP pathway, and enhanced growth performance and immune function in DQ challenged mice. This study provides new insights into the anti-inflammatory potential of plant polysaccharides and their ability to alleviate intestinal injury.

## 1 Introduction

In recent years, intensive farming has become the predominant livestock production system owing to its efficiency, scalability, and standardized management. However, the high stocking densities typical of intensive farming promote pathogen proliferation and the use of management practices such as early weaning, both of which readily induce stress in animals (1, 2). The intestine, the body's largest immune organ, plays a critical role in defending against antigens and pathogens. Prolonged stress compromises the intestinal barrier, triggering inflammatory diseases and potentially causing diarrhea or even death in young animals (3). Antibiotics were traditionally used to promote animal health; however, concerns over residues and antimicrobial resistance have led China to ban their use in feed. Consequently, alleviating animal stress has become a major focus in modern livestock farming.

Allium mongolicum Regel, commonly known as Mongolian garlic, is a wild species of the genus Allium native to the Inner Mongolia region. In recent years, with increasing interest in its nutritional value and functional properties, polysaccharides from Allium mongolicum Regel (AMRP) have been identified as one of its major bioactive components and have been demonstrated to exhibit anti-inflammatory (4), antioxidant (5), and immunomodulatory activities (6), indicating their potential as a green and environmentally friendly feed additive. Dietary supplementation with AMRP has been shown to improve growth performance (7), immune function, and antioxidant capacity (8), as well as meat quality in meat sheep. Similarly, supplementation of AMRP in dairy cow diets has been reported to enhance plasma immune function and antioxidant capacity (9). Taken together, AMRP, as a naturally derived and multifunctional plant polysaccharide, exhibits promising application prospects in ruminant nutrition and health promotion. However, its biological efficacy in different application scenarios may be influenced by factors such as preparation processes, structural characteristics, and underlying mechanisms. Therefore, further optimization of extraction conditions is required to provide a theoretical basis for the development and application of AMRP as a green feed additive. AMRP using conventional hot-water extraction generally suffer from low yields.

At present, plant polysaccharides are mainly extracted using hot-water extraction, alkaline extraction, or ultrasound-assisted extraction methods. However, these traditional approaches are often associated with long extraction times, high energy consumption, and potential structural degradation of polysaccharides under high-temperature conditions, which may ultimately compromise polysaccharide yield and biological activity. In contrast, enzymatic extraction offers several advantages, including high specificity, mild reaction conditions, operational simplicity, and good safety, and has therefore been widely applied in plant polysaccharide extraction studies in recent years (10). Commonly used enzymes include cellulase, papain, and pectinase (11). Accordingly, the present study employed enzymatic extraction to obtain AMRP and optimized the extraction conditions using response surface methodology, with the aim of maximizing the yield and content of AMRP.

The intestine is not only the primary site of digestion and absorption but also the largest immune organ. The intestinal epithelium contains a complex network of endoplasmic reticulum (ER). Stress disrupts the ER lumen, impairing cellular function. This disruption leads to the accumulation of misfolded proteins and triggers the unfolded protein response (UPR). The buildup of misfolded proteins in the ER lumen results in ER stress (ERS) (12). UPR alleviates ERS mainly through three pathways (13): inositol-requiring enzyme 1 (IRE1), protein kinase-like ER kinase (PERK), and activating transcription factor 6 (ATF6) (14, 15). All three pathways are initiated by the ER chaperone glucose-regulated protein 78 (GRP78). UPR mitigates ERS by upregulating molecular chaperones and activating ER folding enzymes (16, 17). Moderate ERS serves as a protective mechanism, but prolonged or severe ERS may overwhelm UPR regulation. This condition can activate mitochondrial autophagy, exacerbate inflammation, and upregulate apoptosis-related genes, ultimately causing cell death (18). During intestinal ERS, GRP78 dissociates from PERK, leading to a rapid increase in GRP78 expression (19). This phosphorylation of PERK activates Eukaryotic Initiation Factor 2 Alpha Subunit (EIF-2α), reducing protein synthesis, and promotes NF-κB nuclear translocation, thereby inducing inflammatory factor release. EIF-2α concurrently induces Activating Transcription Factor 4 (ATF4) translation, and ATF4 activates C/EBP Homologous Protein (CHOP) transcription, leading to CHOP upregulation (20). This, in turn, activates caspase-mediated apoptosis (21). Water extracts of purslane inhibit mitochondrial autophagy by downregulating p-PERK and its downstream autophagy gene Beclin1, thereby alleviating ERS and improving intestinal inflammation (22). Codonopsis polysaccharides alleviate ERS and protect nerve cells by downregulating key genes in the PERK/ATF4/CHOP pathway and their downstream apoptosis-related genes (23). In summary, plant polysaccharides mitigate excessive ERS and downregulate apoptosis-related genes, thereby reducing inflammation. Based on this, we hypothesize that *AMRP* may protect against intestinal damage via the PERK/ATF4/CHOP ERS pathway. DQ (*DQ*) injection impairs intestinal barrier function and morphology and markedly increases mitochondrial reactive oxygen species (ROS) (3). Excessive ROS causes mitochondrial dysfunction and metabolic disturbance, thereby triggering ERS (24, 25). Therefore, this study established a *DQ-*induced mouse model of intestinal injury to investigate whether *AMRP* alleviates intestinal damage by modulating ERS and suppressing inflammatory factor production, thereby providing a theoretical basis for its use as a green feed additive.

## 2 Materials and methods

### 2.1 Materials

Allium mongolicum Regel powder (Cat. No.: HH-SCF-001) was obtained from Haohai Biotechnology Co., Ltd., Shanghai, China (Alxa League, Inner Mongolia). Papain (800 U/mg Cat. No.: S10087), cellulase (400 U/mg Cat. No.: S10068), and anthraquinone (Cat. No.: S10010) were purchased from Shanghai Yuanye Biotechnology Co., Ltd., Shanghai, China. Pectinase (500 U/mg Cat. No.: XY-C1068) was obtained from Shanghai Xinyu Biotechnology Co., Ltd., Shanghai, China. Anhydrous ethanol (analytical grade, Cat. No.: BL0101), anhydrous glucose (analytical grade, Cat. No.: FC1002) and sulfuric acid (analytical grade, Cat. No.: 10021188) were all of analytical grade, among which anhydrous ethanol was supplied by Tianjin Beilian Fine Chemical Development Co., Ltd., Tianjin, China, anhydrous glucose was from Tianjin Fuchen Chemical Reagent Factory, Tianjin, China, and sulfuric acid was purchased from China National Pharmaceutical Group Chemical Reagent Co., Ltd., Shanghai, China. Enzyme-linked immunosorbent assay (ELISA) kits, including immunoglobulin A (IgA, Cat. No.: JYM0560Mo), immunoglobulin M (IgM, Cat. No.: JYM0032Mo), immunoglobulin G (IgG, Cat. No.: JYM0031Mo), interleukin-1β (IL-1β, Cat. No.: JYM0531Mo-T), interleukin-10 (IL-10, Cat. No.: JYM0005Mo), interleukin-4 (IL-4, Cat. No.: JYM0011Mo), interleukin-6 (IL-6, Cat. No.: JYM0012Mo-T) and tumor necrosis factor-α (TNF-α, Cat. No.: JYM0218Mo-T), were purchased from Wuhan Jiyinmei Biotechnology Co., Ltd., Wuhan, China. Total RNA extraction reagent (RNAiso Plus) was obtained from Takara Biomedical Technology (Beijing) Co., Ltd., Beijing, China. The cDNA Synthesis Kit (Cat. No.: AG11711) and quantitative real-time polymerase chain reaction (qRT-PCR) Kit (Cat. No.: AG11763) were both purchased from Hunan Aikerui Bioengineering Co., Ltd., Changsha, China. The primary antibodies against PERK (Cat. No.: 68482-1-Ig), phospho-PERK (p-PERK, Cat. No.: 82534-1-RR), GRP78 (Cat. No.: 11587-1-AP), EIF-2α (Cat. No.: 11170-1-AP), ATF4 (Cat. No.: 10835-1-AP), CHOP (Cat. No.: 15204-1-AP), Bcl-2 (Cat. No.: 68103-1-Ig) and Bax (Cat. No.: 50599-2-Ig) were all acquired from Wuhan Sanying Biotechnology Co., Ltd. (Wuhan, China) Diquat (Cat. No.: 45422-250MG-R) was purchased from Merck KGaA, Darmstadt, Germany.

### 2.2 Methods

#### 2.2.1 Preparation of AMRP

Allium mongolicum Regel powder was purchased from Haohai Biotechnology Co., Ltd., Shanghai, China (Alxa League, Inner Mongolia). *AMRP* was prepared by enzymatic hydrolysis following the method of Yin et al. (26), with slight modifications. Briefly, 10.0 g of powder was weighed and extracted. The sample was mixed with 200 ml of distilled water (material-to-liquid ratio 1:20, g/ml). The mixture was hydrolyzed in a thermostatic shaking incubator at 150 r/min under the specified enzymatic conditions to obtain the *AMRP* extract. The extract was then heat-inactivated at 75 °C and vacuum-filtered. The filtrate was concentrated to 30 ml and precipitated with anhydrous ethanol. The resulting precipitate was freeze-dried to yield *AMRP*.

#### 2.2.2 Screening of enzyme types and reaction conditions

At a material-to-liquid ratio of 1:20, hydrolysis temperature of 55 °C, hydrolysis time of 3.5 h, enzyme addition of 15%, and shaking speed of 150 r/min, papain, cellulase, and pectinase were tested to evaluate their effects on *AMRP* yield. Each treatment was performed in triplicate. Based on the enzyme screening results, further experiments were conducted as follows: with a material-to-liquid ratio of 1:20, hydrolysis temperature of 55 °C, and hydrolysis time fixed at 3.5 h, the effects of varying enzyme addition levels (5%, 10%, 15%, 20%, and 25%) on *AMRP* yield were evaluated. With the hydrolysis time fixed at 2.5 h and enzyme addition at 15%, the effects of different hydrolysis temperatures (45, 50, 55, 60, and 65 °C) on *AMRP* yield were examined. With the hydrolysis temperature fixed at 50 °C and enzyme addition at 15%, the effects of different hydrolysis times (1.5, 2.5, 3.5, 4.5, and 5.5 h) on *AMRP* yield were studied. Each treatment was repeated three times.

Based on the single-factor experiments, response surface methodology (RSM) was applied to optimize *AMRP* extraction conditions. Enzyme addition (A), hydrolysis time (B), and hydrolysis temperature (C) were chosen as the independent variables, with polysaccharide content as the response variable. A Box–Behnken design was used with three levels for each factor. The experimental design and factor levels are shown in Supplementary Table S1.

#### 2.2.3 Determination of sugar content in AMRP

The *AMRP* content was measured using the anthrone-sulfuric acid method (27), with glucose (g/ml) used as the standard. A standard curve for glucose was generated by plotting the absorbance at 625 nm (y-axis) against the glucose concentration (x-axis). The regression equation of the standard curve was y = 8.366x + 0.439, with a correlation coefficient (*R*^2^) of 0.9986.

### 2.3 Animal experiment

Specific pathogen-free (SPF) male mice were obtained from Beijing Spafas Biotechnology Co., Ltd., Beijing, China (license number: SCXK (Meng) 2020–0002). The mice were housed in the animal facility of Inner Mongolia Agricultural University under controlled conditions (22 ± 2 °C, 40–60% humidity, 12 h light/dark cycle). Thirty-six 4-week-old mice (20 ± 2 g) were randomly assigned to three groups: control (CON), Diquat (*DQ*), and DQ-injected group (*AMRP*+*DQ*). Mice were acclimatized for 7 days before a 21-day experimental period. Throughout the experiment, mice had free access to food and water. At 8:00 a.m. daily, mice in the *AMRP*+*DQ* group received 0.2 ml of *AMRP* solution (80 mg/ml) by oral gavage, while the CON and *DQ* groups were gavaged with the same volume of physiological saline. At the end of the experiment, mice were fasted for 12 h before body weight was recorded. Subsequently, mice in the *DQ* and *AMRP*+*DQ* groups were subcutaneously injected with *DQ* solution (24 mg/kg body weight), while the CON group received the same volume of saline. After another 12 h of fasting, the mice were euthanized and samples were collected (28). The nutritional composition of the diet is shown in Supplementary Table S2.

At the end of the experiment, mice were anesthetized with ether, and blood was collected from the orbital sinus into centrifuge tubes. Samples were allowed to stand at room temperature at a 45° angle for 30 min, then centrifuged at 3,500 rpm for 10 min. The supernatant was collected and stored at −80 °C. Mice were euthanized by cervical dislocation, and their limbs were pinned to the surgical table. The abdominal cavity was opened, and 2–3 cm segments of the intestine were excised, excise a portion of the jejunum, rinsed three times with sterile saline, and transferred to cryogenic tubes. Samples were immediately snap-frozen in liquid nitrogen and stored at −80 °C (29).

### 2.4 Organ index

At the end of the experiment, mice were weighed. Twelve hours after *DQ* injection, mice were euthanized by cervical dislocation. The liver, thymus, and spleen were collected, blotted dry, and weighed to calculate organ indices, expressed as the ratio of organ weight to body weight (28).

### 2.5 Enzyme-linked immunosorbent assay (ELISA)

Serum and intestine levels of IL-1β, IL-6, IL-4, IL-10, TNF-α, IgA, IgG, and IgM were measured following the manufacturer's instructions of the ELISA kits Using the standard concentration as the x-axis and the corresponding OD values as the y-axis, standard regression curves were plotted for different inflammatory factors and Immunoglobulin to determine the concentrations of the relevant proteins (30).

### 2.6 Quantitative real-time PCR (RT-qPCR)

Total mRNA was extracted from the intestine of all mice using Trizol reagent and reverse transcribed into cDNA using a reverse transcription kit according to the instruction book. The AG SYBR Green Pro Taq HS was used to analyze the PERK/ATF4/CHOP signaling pathway related genes in mouse tissues by RT-qPCR (31). The sequences of all primers used in this experiment are presented in Supplementary Table S3. GAPDH was used as the reference gene, and the relative mRNA expression levels of different genes were calculated using the 2 ^ΔΔ*CT*^.

### 2.7 Hematoxylin and eosin staining (HE) and immunohistochemical staining (IHC)

The intestine was fixed in 4 % paraformaldehyde and then embedded with paraffin. Hematoxylin and Eosin (H&E) staining was performed according to the standard protocols. The sections were dewaxed and incubated in 3% hydrogen peroxide solution at room temperature in the dark for 25 min (The section thickness was 5 μm). They were then washed three times with PBS (pH 7.4) on a decolorizing shaker, 5 min each wash. Within the hydrophobic barrier, 3% BSA was applied to evenly cover the tissue, and blocking was performed at room temperature for 30 min. Primary antibodies were added and incubated overnight at 4 °C. After three PBS washes, the sections were incubated with secondary antibodies at room temperature for 50 min, followed by three additional PBS washes (5 min each). The sections were gently drained, and chromogenic solution was added until a brown-yellow color indicated positive staining; the reaction was stopped by rinsing with distilled water. Counterstaining was performed with hematoxylin for ~3 min, followed by rinsing with distilled water, bluing in hematoxylin bluing solution, and washing with running water. Finally, the sections were dehydrated. For all the above staining procedures, quantitative analysis of randomly selected regions was performed using ImageJ 1.8.0 (32).

### 2.8 Immunohistochemical analysis of tight junction proteins

Immunohistochemical staining was performed to evaluate the expression of intestinal tight junction proteins, including zonula occludens-1 (ZO-1), Claudin-1, and Occludin. Briefly, intestinal tissue samples were fixed in 4% paraformaldehyde for 24 h, dehydrated through a graded ethanol series, cleared in xylene, and embedded in paraffin. Paraffin blocks were sectioned at a thickness of 4 μm using a rotary microtome and mounted onto glass slides. The sections were deparaffinized in xylene and rehydrated through graded ethanol solutions to distilled water. Antigen retrieval was carried out by heating the sections in citrate buffer (10 mM, pH 6.0). After cooling to room temperature, endogenous peroxidase activity was blocked by incubation with 3% hydrogen peroxide for 10–15 min. The sections were then rinsed with phosphate-buffered saline (PBS, pH 7.4) and blocked with 5% bovine serum albumin (BSA) for 30 min at room temperature to reduce nonspecific binding. Subsequently, the sections were incubated overnight at 4 °C with primary antibodies against ZO-1 (1:1,000), Claudin-1 (1:500), and Occludin (1:1,000; Wuhan Sanying Biotechnology Co., Ltd.). After washing with PBS, the sections were incubated with the appropriate horseradish peroxidase (HRP)-conjugated secondary antibodies for 30–60 min at room temperature. Immunoreactivity was visualized using 3,3′-diaminobenzidine (DAB) as the chromogen, followed by counterstaining with hematoxylin. The stained sections were dehydrated, cleared, and mounted with neutral resin. Images were captured using a light microscope. The expression levels of tight junction proteins were evaluated based on staining intensity and distribution, and semi-quantitative analysis was performed using ImageJ software (33).

### 2.9 Data analysis

Experimental data were compiled and analyzed using Microsoft Excel 2010, followed by one-way ANOVA in SAS 9.2. *Post-hoc* comparisons among groups were performed using Duncan's multiple range test. Data are presented as mean ± SEM. A *P*-value < 0.01 was considered highly significant, *P* < 0.05 significant, and 0.05 < *P* < 0.1 indicative of a trend.

## 3 Results

### 3.1 Impact of enzyme types, dosage, temperature, time on polysaccharide yield

**Figure 1:**
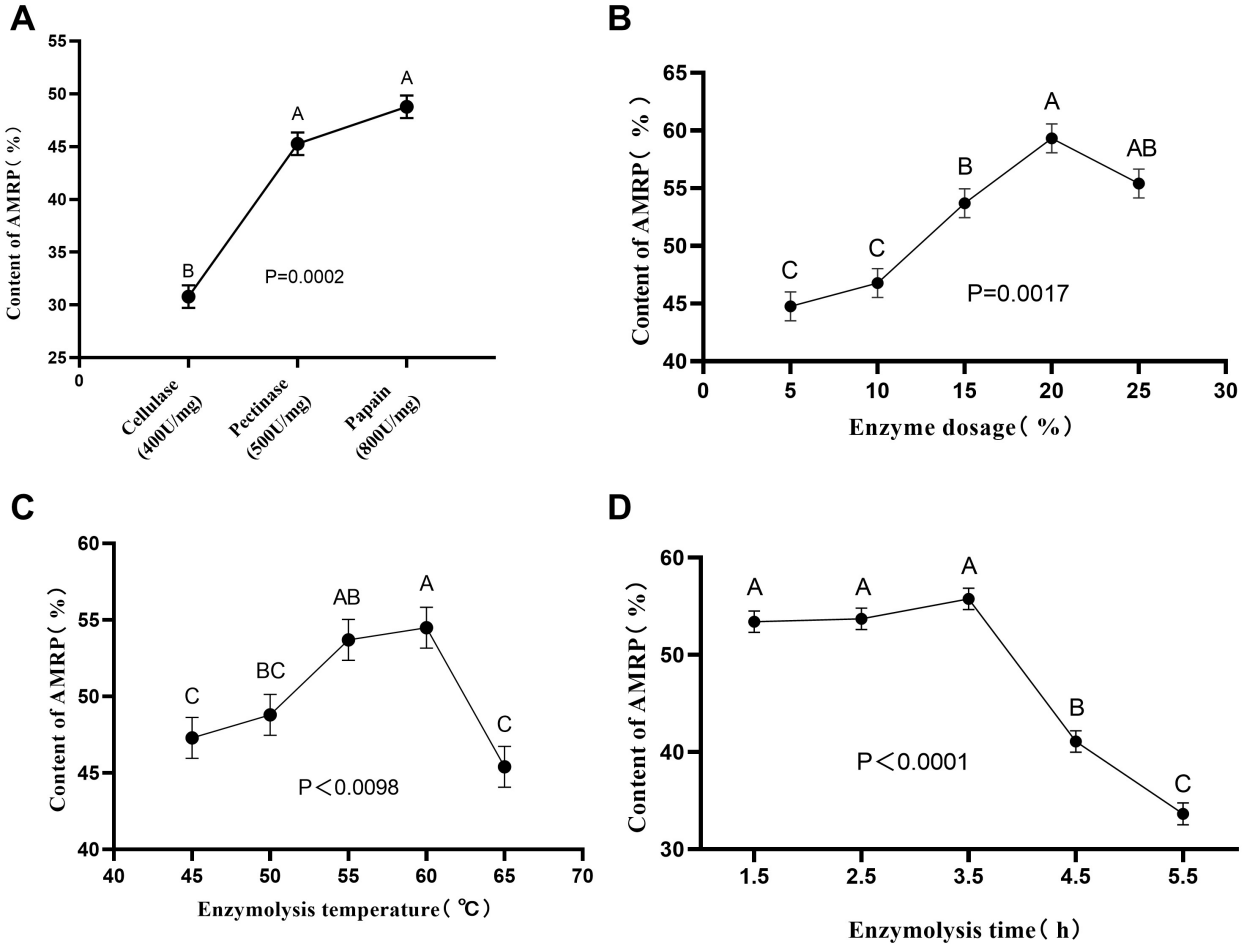
Optimization of *AMRP* extraction conditions. **(A)** Enzyme type; **(B)** Enzyme dosage; **(C)** Hydrolysis temperature; **(D)** Hydrolysis time. (Different letters represent significant differences, *P* < 0.05). (*n* = 3).

As shown in Figure 1A, among *AMRP* extracted with papain, cellulase, and pectinase, the papain-treated group had the highest polysaccharide content, which was significantly higher than that of the cellulase group (*P* < 0.001). Accordingly, papain was chosen for subsequent experiments to examine the effects of enzyme dosage, hydrolysis temperature, and hydrolysis time on *AMRP* yield. Figure 1B shows that when the enzyme dosage reached 20%, *AMRP* content was significantly higher than that of the 5%, 10%, and 15% groups (*P* < 0.05), reaching 59.32%. Further increases in enzyme dosage led to a decline in *AMRP* content. Figure 1C indicates that at a hydrolysis temperature of 60 °C, *AMRP* content was significantly higher than in the 45 °C, 50 °C, and 65 °C groups (*P* < 0.05), reaching 54.5%. Increasing the hydrolysis temperature beyond 60 °C resulted in reduced *AMRP* content. Figure 1D shows that within the hydrolysis time range of 1.5 to 3.5 h, *AMRP* content increased with time, reaching a maximum of 55.77% at 3.5 h. Prolonging the hydrolysis time beyond 3.5 h caused a significant decrease in *AMRP* content (*P* < 0.001). *AMRP* content in the 4.5 h and 5.5 h groups was significantly lower than in the 1.5–3.5 h groups (*P* < 0.001).

### 3.2 Results of response surface methodology

Based on the single-factor experiment results, enzyme dosage (A), hydrolysis time (B), and hydrolysis temperature (C) were chosen as independent variables, with *AMRP* content (Y) as the response variable.

A three-factor, three-level experiment was designed using the Box-Behnken method, with the results presented in Supplementary Table S4. The results in Supplementary Table S4. were fitted with a quadratic regression, yielding the following equation for *AMRP* content: Y = 61.11 + 4.68A + 0.92B + 0.91C + 1.13AB + 0.90AC + 0.54BC – 2.56A^2^ + 0.18B^2^ – 1.72C^2^. Analysis of variance (ANOVA) was performed on the data in Supplementary Table S4 using Design-Expert 10 software, and the detailed results are summarized in Table 1. The results showed that the model was highly significant (*P* < 0.01), indicating that the regression equation accurately reflected the experimental data and could be used to guide the design and optimization of the *AMRP* extraction process. Moreover, the lack-of-fit *P*-value was 0.2414 (>0.05), indicating no significant lack-of-fit and confirming that experimental errors were minimal, with model residuals mainly due to random variation. Furthermore, the coefficient of determination (*R*^2^) was 0.9764, the adjusted *R*^2^ (*R*^2^Adj) was 0.9461, and the predicted *R*^2^ (*R*^2^Pred) was 0.7544; the differences among these values were less than 0.2, indicating a strong correlation between observed and predicted values. Based on F-values and *P*-values, the factors affecting *AMRP* content were ranked as follows: enzyme dosage (A) > hydrolysis time (B) > hydrolysis temperature (C), with a significant interaction between enzyme dosage and hydrolysis time (*P* < 0.05).

**Table 1:** Analysis of Variance of the regression model.

**Source**	**Sum of squares**	**df**	**Mean square**	***F*-value**	***P*-value**
model	240.47	9	26.72	32.19	< 0.0001
A–The enzyme dosage	175.41	1	175.41	211.35	< 0.0001
B–hydrolysis time	6.77	1	6.77	8.16	0.0245
C–hydrolysis temperature	6.66	1	6.66	8.03	0.0253
AB	5.11	1	5.11	6.15	0.0422
AC	3.2	1	3.2	3.86	0.0902
BC	1.14	1	1.14	1.38	0.2786
A^2^	27.57	1	27.57	33.22	0.0007
B^2^	0.14	1	0.14	0.17	0.6957
C^2^	12.51	1	12.51	15.08	0.006
residual	5.81	7	0.83		
lack of fit	3.56	3	1.19	2.11	0.2414
pure error	2.25	4	0.56		
total deviation	246.28	16			

### 3.3 Validation experiment

The optimal extraction conditions determined by response surface methodology were: enzyme dosage 25.00%, hydrolysis time 4.5 h, and hydrolysis temperature 63.4 °C. For practical feasibility, the experimental conditions were adjusted to an enzyme dosage of 25%, hydrolysis time of 4.5 h, and hydrolysis temperature of 63 °C. Under these conditions, the experiment was conducted in triplicate, yielding an average *AMRP* content of 65.89%. The relative deviation from the theoretically predicted value of 66.26% was 1.18%, confirming the reliability of the parameters obtained from the response surface optimization.

### 3.4 AMRP improves growth performance and organ indices in diquat-challenged mice

**Figure 2:**
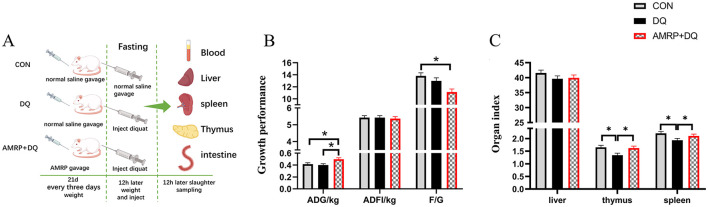
*AMRP* improved growth performance and immune organ indices in mice. **(A)** Animal experiment process. **(B)** Growth performance (ADG: Average Daily Gain. ADFI: Average Daily Feed Intake. F/G: Feed/Gain Ratio) **(C)** Organ indices. (“^*^” indicates significant differences *P* < 0.05, and “^**^” indicates highly significant differences *P* < 0.01). (*n* = 10).

As shown in Figure 2A, no significant differences were observed in average daily gain (ADG), average daily feed intake (ADFI), or feed-to-gain ratio (F/G) between the *DQ* and CON groups. Compared with the *DQ* group, the *AMRP*+*DQ* group showed a significant increase in ADG (*P* < 0.05), whereas ADFI and F/G remained unchanged. Compared with the CON group, the *AMRP*+*DQ* group demonstrated a significant increase in ADG (*P* < 0.05) and a significant reduction in F/G (*P* < 0.05).

As shown in Figure 2B, the thymus and spleen indices of the *DQ* group were significantly lower than those of the CON group (*P* < 0.05). Compared with the *DQ* group, the *AMRP*+*DQ* group displayed significantly higher thymus and spleen indices (*P* < 0.05). No significant differences were observed in thymus and spleen indices between the *AMRP*+*DQ* and CON groups group.

### 3.5 AMRP improve the immune function of mice challenged with diquat

**Figure 3:**
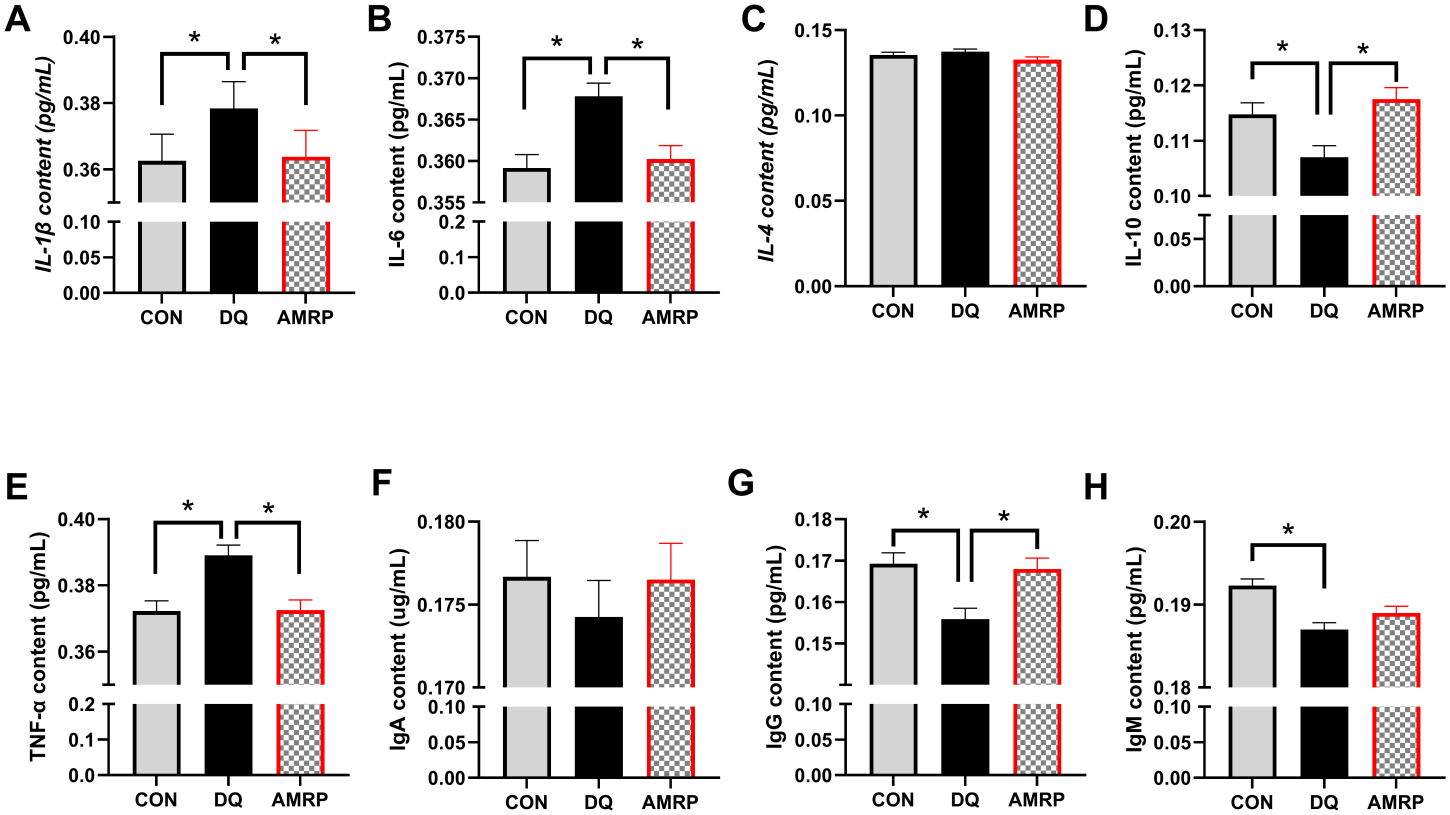
*AMRP* reduced the levels of pro-inflammatory cytokines and increased the levels of anti-inflammatory cytokines in the blood of *DQ*-challenged mice. **(A-C)** Pro-inflammatory cytokine levels in the blood: **(A)** IL-1β; **(B)** IL-6; **(C)** TNF-α; **(D, E)** Anti-inflammatory cytokine levels in the blood: **(D)** IL-10; **(E)** IL-4. **(F-H)** Immunoglobulin levels in the blood: **(F)** IgA; **(G)** IgG; **(H)** IgM. (“^*^” indicates significant differences *P* < 0.05), (*n* = 10).

As shown in Figure 3, compared with the CON group, the *DQ* group exhibited significantly higher concentrations of pro-inflammatory cytokines IL-1β, IL-6, and TNF-α (*P* < 0.05), whereas the levels of IL-10, IgG, and IgM were significantly lower (*P* < 0.05). No significant differences were observed in IL-4 and IgA levels between the *DQ* and CON groups. Compared with the *DQ* group, the *AMRP*+*DQ* group showed significantly reduced concentrations of IL-1β, IL-6, and TNF-α (*P* < 0.05), while IL-10 and IgG levels were significantly elevated (*P* < 0.05). Compared with the CON group, the *AMRP*+*DQ* group showed no significant differences in the levels of IL-1β, IL-6, IL-4, IL-10, TNF-α. IgA, IgG or IgM.

### 3.6 AMRP alleviate intestinal injury and restore function in mice challenged with diquat

**Figure 4:**
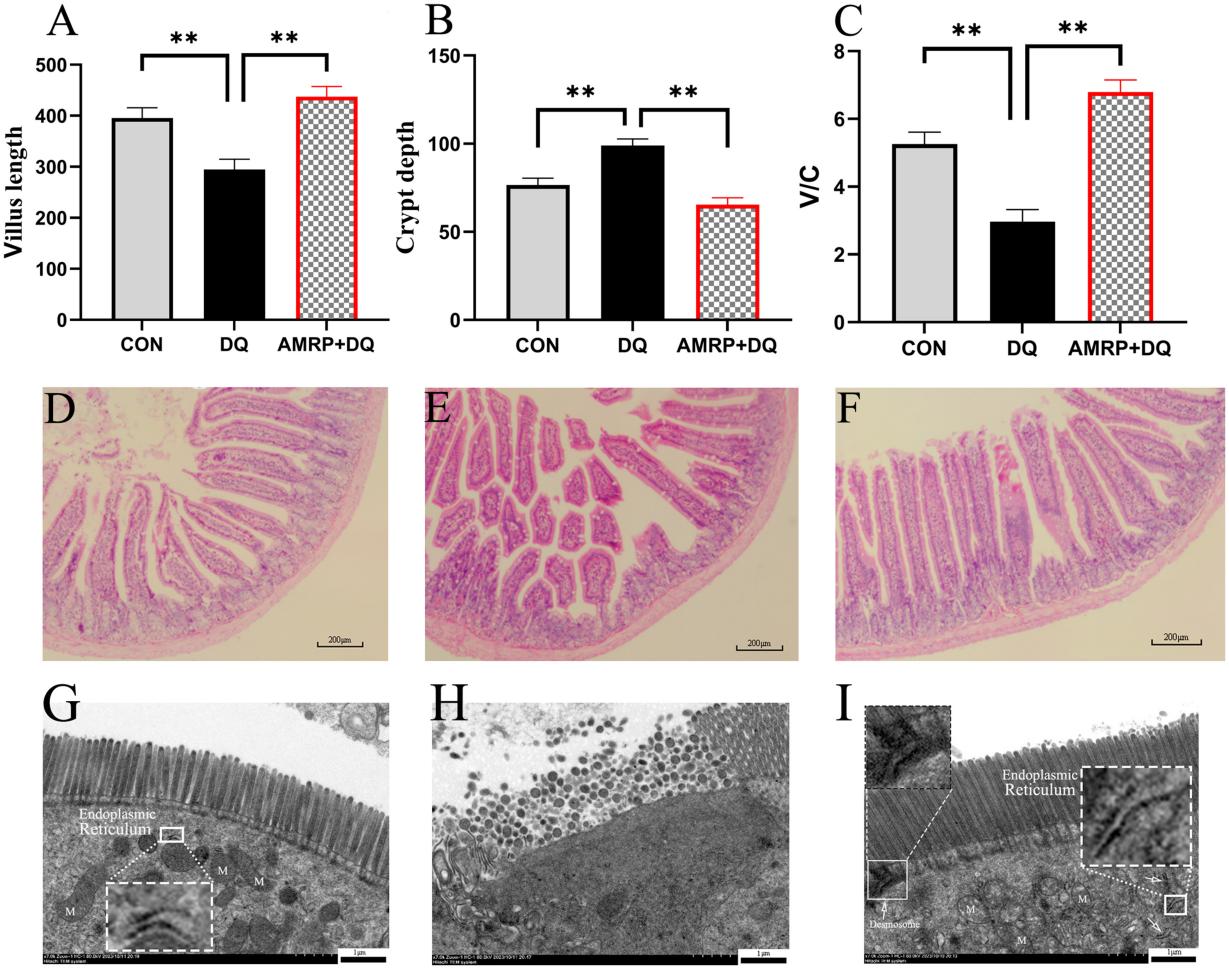
*AMRP* improved intestinal morphology in mice. **(A-C)** Villus length, crypt depth, and villus-to-crypt ratio. **(D-F)** Representative H&E-stained sections of the intestine from different treatment groups: **(D)** CON group, **(E)** *DQ* group, and **(F)** *AMRP*+*DQ* group (scale bar = 200 μm). **(G-I)** Ultrastructural electron microscopy images of intestinal tissues of mice from different treatment groups: **(G)** CON group, **(H)** *DQ* group, and **(I)** *AMRP*+*DQ* group (ultrastructure 7000 ×, scale bar = 1μm), “M”: Mitochondria; White arrow: ER (endoplasmic reticulum). (“^*^” indicates significant differences *P* < 0.05, and “**” indicates highly significant differences *P* < 0.001), (*n* = 10).

As shown in Figures 4A–C, compared with the CON group, the *DQ* group exhibited a significantly reduced small intestinal villus height (*P* < 0.01), a significantly increased crypt depth (*P* < 0.01), and a significantly decreased villus height-to-crypt depth ratio (*P* < 0.01). Compared with the *DQ* group, the *AMRP*+*DQ* group showed a significant increase in villus height (*P* < 0.01), a significant decrease in crypt depth (*P* < 0.01), and a significant increase in the villus height-to-crypt depth ratio (*P* < 0.01). These results indicate that *DQ* induced small intestinal damage in mice, confirming successful model establishment, while dietary supplementation with *AMRP* effectively alleviated *DQ*-induced intestinal mucosal injury.

As shown in Figures 4D–F, the small intestinal villi in the CON group were slender, orderly, and densely *p*acked. In contrast, the *DQ* group displayed sparse villi with evident breakage, swelling, and shortening. Compared with the *DQ* group, the *AMRP*+*DQ* group exhibited markedly improved villus morphology, characterized by longer, more closely arranged villi, reduced villus breakage and shedding, and narrower inter-villus spaces. As shown in Figures 4G–I, the small intestinal epithelial microvilli in the CON group were smooth, orderly, and associated with normally shaped mitochondria. Compared with the CON group, the *DQ* group exhibited sparse, disorganized microvilli of irregular length and a marked reduction in mitochondria. Compared with the *DQ* group, the *AMRP*+*DQ* group displayed an increased number of mitochondria, more orderly microvilli, and clearly visible intercellular desmosomes.

### 3.7 AMRP reduces intestinal pro-inflammatory cytokine production in diquat-challenged mice

**Figure 5:**
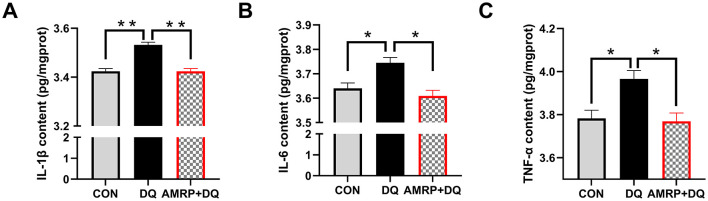
AMRP reduced intestinal pro-inflammatory cytokine levels in DQ-challenged mice. **(A)** IL-1β; **(B)** IL-6; **(C)** TNF-α. (“^*^” indicates significant differences *P* < 0.05, and “^**^” indicates highly significant differences *P* < 0.01), (*n* = 10).

As shown in Figure 5, the *DQ* group exhibited significantly elevated IL-1β levels (*P* < 0.01) and significantly increased IL-6 and TNF-α levels (*P* < 0.05) compared with the CON group. In contrast, the *AMRP*+*DQ* group showed significantly reduced IL-1β levels (*P* < 0.01) and decreased IL-6 and TNF-α levels (*P* < 0.05) compared with the *DQ* group. No significant differences in IL-1β, IL-6, or TNF-α levels were observed between the *AMRP*+*DQ* and CON groups.

### 3.8 AMRP inhibits the PERK/ATF4/CHOP signaling pathway in DQ-challenged

**Figure 6:**
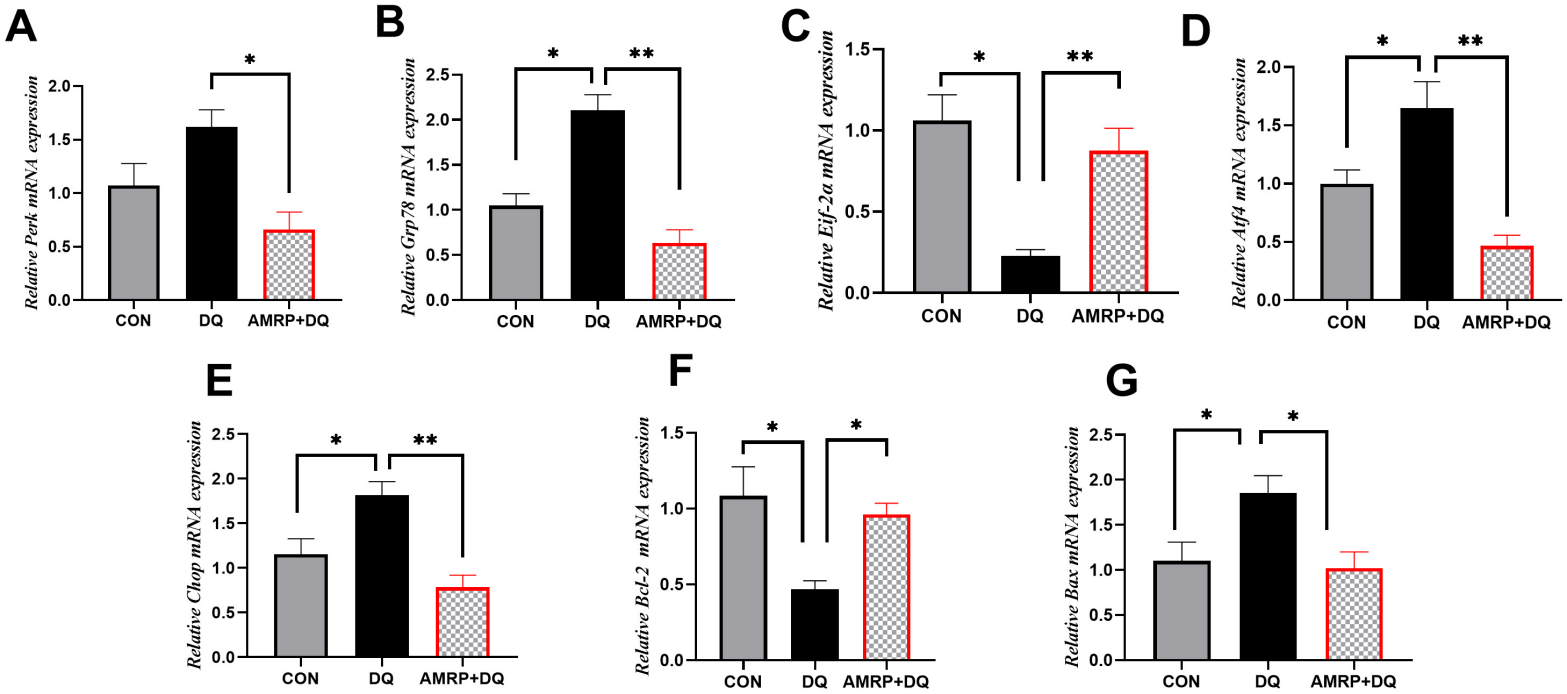
*AMRP* inhibited excessive activation of the endoplasmic reticulum stress (ERS) pathway PERK/ATF4/CHOP in *DQ*-challenged mice. **(A-H)** Relative mRNA expression levels of key ERS pathway genes: **(A)** Perk; **(B)** Grp78; **(C)** Eif-2α; **(D)** Atf4; **(E)** Chop; **(F)** Bcl-2; **(G)** Bax. (“^*^” indicates significant differences *P* < 0.05, and “^**^” indicates highly significant differences *P* < 0.01), (*n* = 10).

As shown in Figure 6, the *DQ* group displayed significantly increased mRNA expression of Grp78, Atf4, Chop, and Bax (*P* < 0.05) and significantly decreased expression of Eif-2α and Bcl-2 (*P* < 0.05) compared with the CON group, whereas PERK expression levels remained unchanged. Compared with the *DQ* group, the *AMRP*+*DQ* group exhibited significantly reduced Perk and Bax expression (*P* < 0.05) and markedly decreased Grp78, Atf4, and Chop expression (*P* < 0.01). Additionally, Eif-2α expression was significantly elevated (*P* < 0.01), and Bcl-2 expression was increased (*P* < 0.05) in the *AMRP*+*DQ* group. No significant differences in these parameters were observed between the *AMRP*+*DQ* and CON groups.

**Figure 7:**
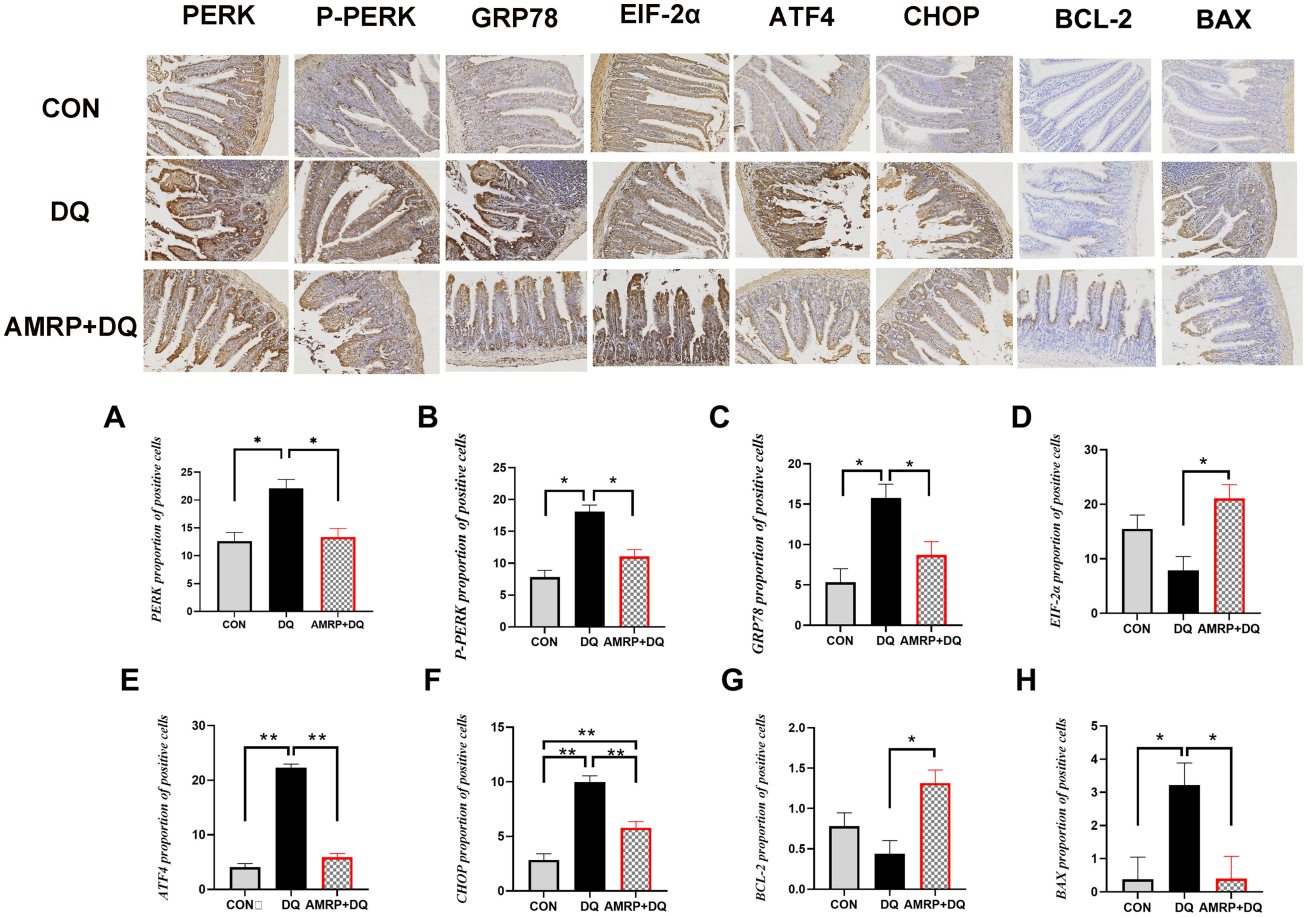
Effects of *AMRP* on protein expression levels of key molecules in the endoplasmic reticulum stress (ERS) pathway. **(A-H)** Protein expression levels in each treatment group: **(A)** PERK; **(B)** p-PERK; **(C)** GRP78; **(D)** EIF-2α; **(E)** ATF4; **(F)** CHOP; **(G)** BCL-2; **(H)** BAX. (“^*^” indicates significant differences *P* < 0.05, and “^**^” indicates highly significant differences *P* < 0.01), (*n* = 10).

As shown in Figure 7, the *DQ* group displayed markedly elevated protein levels of ATF4 and CHOP (*P* < 0.01) and significantly increased levels of PERK, p-PERK, GRP78, and BAX (*P* < 0.05) compared with the CON group, whereas EIF-2α and BCL-2 levels showed no significant changes. Compared with the *DQ* group, the *AMRP*+*DQ* group exhibited significantly reduced p-PERK, GRP78, and BAX levels (*P* < 0.05) and markedly decreased ATF4 and CHOP levels (*P* < 0.01), while EIF-2α and Bcl-2 levels were significantly elevated (*P* < 0.05). Compared with the CON group, the *AMRP*+*DQ* group showed a significantly higher CHOP protein level (*P* < 0.01), while the levels of PERK, ATF4, p-PERK, GRP78, BCL-2, and BAX did not differ significantly.

## 4 Discussion

Allium mongolicum Regel polysaccharides (AMRP) are the major bioactive components of Allium mongolicum Regel and have been reported to possess multiple biological activities, including immunomodulatory, antioxidant, and anti-inflammatory effects (4–6). However, the extraction process has, to some extent, limited the yield of AMRP and subsequent functional investigations. Traditional hot-water extraction is simple to operate but is generally associated with low extraction efficiency and long processing times. In contrast, enzymatic extraction, owing to its high specificity and efficiency, can degrade structural components of the cell wall such as cellulose and pectin, thereby disrupting the physical barriers that restrict polysaccharide release and significantly improving extraction efficiency and yield (34). Moreover, enzymatic extraction can simultaneously degrade certain impurities, facilitating downstream separation and purification processes, and is therefore widely applied in the extraction of plant polysaccharides (35). Previous studies have demonstrated that, compared with conventional extraction methods, enzymatic extraction can significantly enhance the sugar content and yield of pumpkin seeds polysaccharides (11), and similar advantages have been reported for polysaccharides extracted from Dendrobium Officinale, Vietnamese Red Ganoderma lucidum, and Pleurotus eryngii (36–38). These findings provide a theoretical basis for the application of enzymatic extraction in the preparation of plant polysaccharides. Based on this background, the present study employed an enzyme-assisted ethanol precipitation method to optimize the extraction process of AMRP and compared the extraction efficiencies of different enzyme preparations. The results showed that, under identical conditions, papain treatment yielded a higher AMRP content than cellulase or pectinase treatment, suggesting that proteases may enhance polysaccharide release by degrading cell structure–associated proteins. On this basis, the effects of hydrolysis time, temperature, and enzyme dosage on AMRP yield were further investigated, and the extraction conditions were optimized using response surface methodology. Under the optimized conditions (enzyme dosage 25%, hydrolysis time 4.5 h, and temperature 63.4 °C), the yield of AMRP reached 66.26%. Previous studies have also reported that AMRP obtained via enzymatic extraction exhibits strong *in vitro* antioxidant activity (Figure 1). Compared with polysaccharides obtained using our previously employed hot water extraction-alcohol precipitation method, the *AMRP* yield was increased; however, its bioactivity remains to be further investigated (39).

Dietary supplementation with Allium mongolicum Regel powder and its extracts can enhance growth performance in sheep by increasing average daily gain (ADG) and reducing the feed-to-gain ratio (40). Previous studies have demonstrated that adding ginseng, astragalus, and salvia polysaccharides to the diet increases both average daily gain and average daily feed intake in broilers (41). Furthermore, a study in ruminants indicate that fermented wheat bran polysaccharides promote rumen development and enhance growth performance (42). These results suggest that polysaccharides, the primary bioactive components of plant extracts, can improve animal growth performance. In this study, *AMRP* supplementation significantly increased ADG and reduced the feed-to-gain ratio compared with the control group, improved feed conversion efficiency thereby growth performance in mice (Figure 2B). Previous research has shown that intraperitoneal injection of *DQ* induces intestinal mucosal damage in piglets (3). Mucosal damage increases intestinal permeability, allowing bacteria to invade the lamina propria, which can damage immune organs such as mesenteric lymph nodes and the spleen and trigger abnormal immune responses (43). The thymus and spleen are key immune organs, and their indices directly reflect the animal's immune capacity (44). Ginseng polysaccharides have been shown to enhance immune function in mice by restoring damaged immune organs, increasing their relative weight, reducing inflammatory cytokine production, and promoting immunoglobulin secretion (45). In this study, thymus and spleen indices were significantly lower in the *DQ* group than in the CON group, with varying degrees of atrophy, indicating that *DQ* damaged the mice's immune organs. Conversely, *AMRP* supplementation significantly increased thymus and spleen indices in *DQ*-challenged mice (Figure 2C). *AMRP* also significantly reduced pro-inflammatory cytokines (IL-1β, IL-6, and TNF-α) and increased IL-10 and IgG levels in the blood of *DQ*-challenged mice (Figure 3). The thymus is responsible for T lymphocyte development and maturation (46), while the spleen contains diverse immune cells and factors, serving as a key site for immune responses and playing a crucial role in inflammation (47). These findings suggest that *AMRP* enhances thymus and spleen immune function in mice, thereby improving overall immune capacity and mitigating *DQ*-induced declines in growth performance.

The intestine functions both as the primary site for nutrient absorption and as the body's largest immune organ. Being directly exposed to the external environment, the intestine is vulnerable to damage from harmful factors, including reactive oxygen species, toxins, and hyperosmotic stress, which can trigger severe inflammation, autoimmune disorders, metabolic dysfunctions, and organ pathologies (48). Studies have shown that polysaccharides from Abelmoschus manihot alleviate intestinal inflammation in mice by reducing pro-inflammatory cytokines (TNF-α and IL-6) and promoting the repair of colonic epithelial cells (49) thereby preventing colitis-induced intestinal damage. Polysaccharides from Chinese yam and Cyclocarya paliurus have been shown to strengthen the intestinal barrier by upregulating tight junction proteins and reducing inflammatory cytokines, significantly alleviating dextran sulfate sodium (DSS)-induced ulcerative colitis in mice (50). Together, these findings suggest that plant polysaccharides enhance intestinal immune and anti-inflammatory functions, mitigating inflammation-induced intestinal damage. Based on these findings, we established a mouse model of intestinal injury via intraperitoneal *DQ* injection to examine the protective effects of *AMRP* against *DQ*-induced intestinal epithelial damage. Our results showed that *DQ* severely damaged intestinal villi, whereas *AMRP* supplementation markedly improved villus morphology, increased villus length, and significantly elevated the villus-to-crypt ratio in the *AMRP*+*DQ* group compared with the *DQ* group. Electron microscopy revealed that tight junctions between small intestinal epithelial cells were dense and continuous in the CON group, while they appeared loose and sparse in the *DQ* group. In the *AMRP*+*DQ* group, tight junction damage was alleviated, and desmosomal structures were clearly visible between cells. These findings indicate that *AMRP* promotes the development of intestinal villi and microvilli while mitigating *DQ*-induced intestinal damage in mice (Figure 4). Additionally, *AMRP* supplementation significantly reduced intestinal levels of pro-inflammatory cytokines (IL-1β, IL-6, and TNF-α) in *DQ*-challenged mice, suggesting that *AMRP* alleviates intestinal inflammation by inhibiting cytokine production and thus improves intestinal morphology (Figure 5). However, the precise mechanisms through which *AMRP* enhances intestinal morphology remain unclear.

The intestinal mucosal epithelium is rich in mitochondria, and stressors such as early weaning, heat exposure, or suboptimal feeding management can lead to the accumulation of unfolded proteins, thereby inducing endoplasmic reticulum stress (ERS) in the intestine. Moderate ERS functions as a protective mechanism; however, prolonged or excessive ERS disrupts the secretion of proteins, including tight junction proteins and mucins, and activates apoptotic pathways, directly damaging the intestine (16). During ERS, GRP78 dissociates from PERK, rapidly increasing GRP78 levels, which aids proper protein folding and the processing of misfolded proteins (19). At the same time, PERK phosphorylation activates downstream EIF-2α, suppressing protein synthesis and decreasing IκB levels, which allows NF-κB nuclear translocation and activation, promoting inflammatory cytokine release. Meanwhile, EIF-2α induces ATF4 expression, and ATF4 translocates to the nucleus to bind the CHOP promoter, upregulating CHOP expression (20). Studies have shown that ginseng and astragalus polysaccharides can attenuate excessive ERS and decrease cell apoptosis by downregulating key transcription factors in the PERK/ATF4/CHOP signaling pathway, thereby mitigating inflammation-induced organ damage (45, 51). Therefore, a mouse intestinal injury model was employed to investigate whether *AMRP* could alleviate *DQ*-induced intestinal damage by modulating the ERS pathway (PERK/ATF4/CHOP). The results demonstrated that DQ challenge significantly upregulated intestinal GRP78 mRNA expression in mice. Although PERK mRNA expression did not differ significantly from that of the CON group, it exhibited an increasing trend. In contrast, the protein expression levels of PERK, (p-PERK), and GRP78 were markedly elevated in the intestines of DQ-treated mice compared with those of the CON group (Figures 6, 7). These findings suggest that PERK dissociated from GRP78 and subsequently underwent dimerization and autophosphorylation, thereby acquiring kinase activity, which may explain the increased p-PERK protein levels. Upon activation, PERK catalyzes the phosphorylation of eIF2α, and phosphorylated eIF2α binds tightly to eukaryotic translation initiation factor 2B, leading to inhibition of translation initiation and a reduction in global protein synthesis (52, 55). Nevertheless, eIF2α phosphorylation selectively promotes ATF4 translation, as ATF4 mRNA can be translated via a non-canonical initiation mechanism that circumvents eIF2α-mediated translational repression. Following nuclear translocation, ATF4 induces the expression of the pro-apoptotic transcription factor CHOP. ATF4 and CHOP further cooperate to activate the transcription of genes associated with the UPR (41, 42). Consistently, the present study showed that the mRNA expression levels of ATF4 and CHOP were significantly increased, accompanied by elevated levels of intestinal inflammatory cytokines, indicating the occurrence of severe endoplasmic reticulum stress in the intestine, which further aggravated intestinal inflammatory responses. Following AMRP intervention, the mRNA and protein expression levels of these markers in the intestines of DQ-challenged mice were significantly reduced, and intestinal inflammatory cytokine levels were concomitantly decreased (Figures 5–7). Notably, when cellular proteostasis is restored and endoplasmic reticulum stress is alleviated, ATF4 and CHOP induce the transcription of growth arrest and DNA damage-inducible protein 34 (GADD34). GADD34 functions as a regulatory subunit of protein phosphatase 1 and catalyzes the dephosphorylation of phosphorylated EIF2α (53). In the present study, compared with the CON group, EIF2α mRNA expression was significantly decreased in the DQ group, and its protein level also showed a downward trend. This phenomenon may be attributed to excessive endoplasmic reticulum stress induced by DQ challenge, which disrupted cellular homeostasis and impaired self-repair capacity, thereby preventing the dephosphorylation of p-EIF2α (Figures 6, 7). In contrast, this alteration was alleviated by AMRP intervention, indicating that AMRP attenuates excessive endoplasmic reticulum stress by inhibiting the PERK/ATF4/CHOP signaling pathway, thereby mitigating inflammation-mediated intestinal injury. The BCL-2 family protein BAX is a major regulator of physiological and pathological cell death. BCL-2 is an anti-apoptotic protein localized to the outer mitochondrial membrane, where it directly binds to the pro-apoptotic protein BAX to form heterodimers. This interaction inhibits BAX conformational activation, mitochondrial translocation, and oligomerization, thereby maintaining mitochondrial membrane integrity. In contrast, BAX is a pro-apoptotic protein that exists in the cytoplasm as an inactive monomer or dimer under resting conditions. Upon activation, BAX translocates to the mitochondria and undergoes oligomerization, forming mitochondrial outer membrane permeabilization (MOMP) pores. This process facilitates the release of cytochrome c and other pro-apoptotic factors from the mitochondria into the cytoplasm, ultimately triggering the caspase cascade (54). As a transcription factor, CHOP can directly bind to the promoter region of the *Bcl-2* gene and suppress the transcription and synthesis of the anti-apoptotic gene *Bcl-2*, thereby reducing intracellular levels of anti-apoptotic proteins and weakening their inhibitory constraint on BAX (21). In the present study, compared with the CON group, both mRNA and protein expression levels of Bcl-2 were significantly decreased in the intestines of DQ-treated mice, whereas the mRNA and protein expression levels of BAX were markedly increased (Figures 6, 7). This alteration is likely attributable to excessive CHOP expression induced by DQ challenge, which suppressed Bcl-2 transcription and synthesis. Reduced BCL-2 expression subsequently impaired its ability to effectively bind inactive cytosolic BAX, resulting in the release of BAX from heterodimeric complexes and its conversion into an activatable monomeric form. Consequently, the imbalance in the Bcl-2/BAX ratio indicates that DQ challenge induces severe endoplasmic reticulum stress in the intestine, leading to excessive activation of mitochondrial autophagy and ultimately triggering apoptosis. In contrast, compared with the DQ group, mice in the AMRP+DQ group exhibited significantly reduced mRNA and protein expression levels of BAX, accompanied by a marked increase in Bcl-2 mRNA and protein expression. This shift effectively alleviated mitochondrial autophagy triggered by the imbalance between Bcl-2 and BAX (Figures 6, 7). Transmission electron microscopy further demonstrated that intestinal tissues from DQ-treated mice displayed sparse mitochondria with swollen morphology and disrupted cristae. Conversely, mitochondria in the intestines of AMRP+DQ-treated mice were more abundant, morphologically intact, and characterized by smooth outer membranes, closely resembling those observed in the CON group (Figure 4). Collectively, these results indicate that AMRP alleviates endoplasmic reticulum stress–induced mitochondrial autophagy by suppressing the PERK/ATF4/CHOP signaling pathway, thereby reducing apoptosis and mitigating inflammation-mediated intestinal injury.

## 5 Conclusion

**Figure 8:**
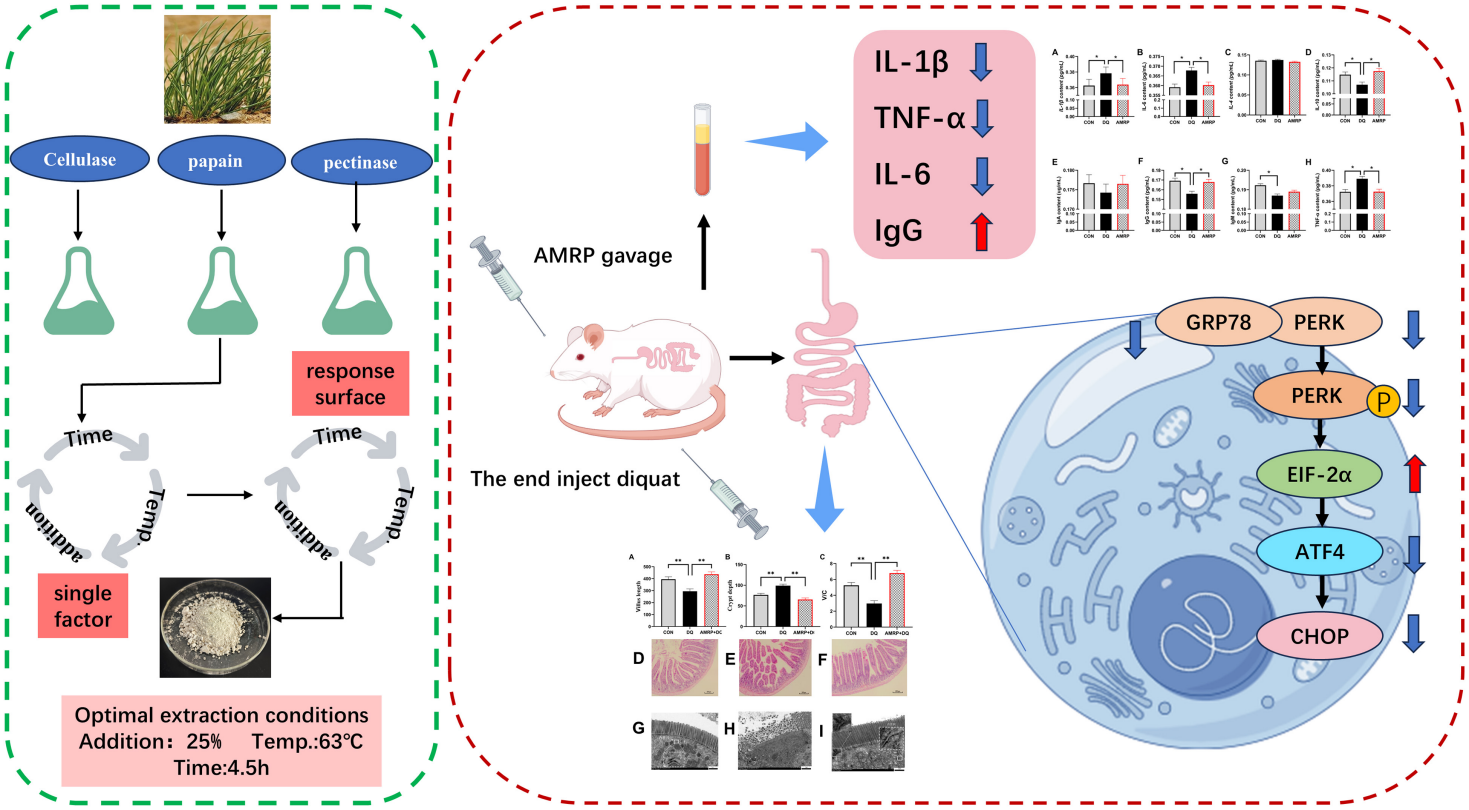
Optimization extraction of Allium mongolicum Regel polysaccharide and alleviation of intestinal injury via inhibition of the PERK/ATF4/CHOP signaling pathway.

In conclusion, *AMRP* mitigates sustained ERS by inhibiting the PERK/ATF4/CHOP pathway, reducing intestinal inflammatory cytokine production, suppressing mitochondrial autophagy, and downregulating apoptotic factors, thereby alleviating *DQ*-induced intestinal injury in mice. Consequently, *AMRP* enhances immune function and improves growth performance (Figure 8).

## Data Availability

The original contributions presented in the study are included in the article/Supplementary material, further inquiries can be directed to the corresponding authors.
