# Correction: Optimization extraction of Allium mongolicum Regel polysaccharide and alleviation of intestinal injury via inhibition of the PERK/ATF4/CHOP signaling pathway

**DOI:** 10.3389/fvets.2026.1864089

**Published:** 2026-05-19

**Authors:** Xiaoyu Niu, Guorong Nan, Yankai Zheng, Yu Chen, Huinan Jiang, Jing Zhang, Lu Chen, Yuanyuan Xing, Dabiao Li

**Affiliations:** 1 College of Animal Science, Inner Mongolia Agricultural University, Hohhot, China; 2 Key Laboratory of Animal Nutrition and Feed Science at Universities of Inner Mongolia Autonomous Region, Hohhot, China; 3 Intellectual Property Protection Center of Inner Mongolia Autonomous Region, Hohhot, China; 4 Animal Husbandry and Veterinary Department, Shanxi Animal Husbandry and Veterinary School, Taiyuan, China

**Keywords:** Allium mongolicum Regel polysaccharides, diquat, endoplasmic reticulum stress, immunity, mitochondrial autophagy

The funder Inner Mongolia Education Department Special Research Project for First Class Disciplines, Project NO. YLXKZX-NND-018 to Yuanyuan Xing was erroneously omitted.

The corrected **Funding** statement is below:

“The author(s) declared that financial support was received for this work and/or its publication. This work was supported by National Natural Science Foundation of Inner Mongolia (Project NO. 2023QN03014); Inner Mongolia Education Department Special Research Project For First Class Disciplines (Project NO. YLXKZX-NND-018).”

The original version of this article has been updated.

