# Supplementary material for: Optimization extraction of Allium mongolicum Regel polysaccharide and alleviation of intestinal injury via inhibition of the PERK/ATF4/CHOP signaling pathway
Source: Front Vet Sci. 2026 Mar 26;13:1773978. doi: 10.3389/fvets.2026.1773978 (PMC13061704; doi:10.3389/fvets.2026.1773978)
Supplement: Supplementary file 1 [file Table_1.docx]

**Supplementary Table S1.**

Levels and factors of response surface design

| factor | level | | |
| --- | --- | --- | --- |
|  | -1 | 0 | 1 |
| A:Enzyme addition (%) | 15 | 20 | 25 |
| B:hydrolysis time（h） | 2.5 | 3.5 | 4.5 |
| C: hydrolysis temperature (℃) | 55 | 60 | 65 |

**Supplementary Table S2.**

Nutrient composition table of mouse diet

| Nutrient level | Content (g/Kg) |
| --- | --- |
| Moisture | 94.1 |
| Crude protein | 209 |
| Crude fat | 49 |
| Crude fiber | 21 |
| Crude ash | 54 |
| Calcium | 12 |
| Total phosphorus | 7.7 |
| Calcium: Total phosphorus | 1.6：1 |
| Lysine | 16.9 |
| Methionine + Cystine | 10.8 |

**Supplementary Table S3.**

The primer sequences for RT-qPCR.

| Primer name | Primer sequences (5'-3') | Gene Bank No. | length (bp) |
| --- | --- | --- | --- |
| GAPDH | forward:TGGTGAAGCAGGCATCTGAG | NM_001289726.2 | 78 |
|  | reverse:TGAAGTCGCAGGAGACAACC |  |  |
| GRP78 | forword:ATTGGAGGTGGGCAAACCAA | NM_001163434.1 | 150 |
|  | reverse:TCGCTGGGCATCATTGAAGT |  |  |
| PERK | forward:TGGATGCCGAGAATGATGGG | NM_001313918.1 | 83 |
|  | reverse:TGGCTTGCTGAGGCTAGATG |  |  |
| EIF-2α | forward:TGACAACAACGACCCTGGAG | NM_026114.3 | 126 |
|  | reverseTGACCACTTTGGGCTCCATC |  |  |
| ATF4 | forward:GAGCTCTTGACCACGTTGGA | NM_001287180.1 | 363 |
|  | reverse:AGAGCCCAGGTAGGACTCTG |  |  |
| CHOP | forward:TCCCCAGGAAACGAAGAGGA | NM_001290183.2 | 106 |
|  | reverse:ATGTGCGTGTGACCTCTGTT |  |  |
| Bcl-2 | forward:GGATAACGGAGGCTGGGATG | NM_177410.3 | 97 |
|  | reverse:GCTGAGCAGGGTCTTCAGAG |  |  |
| Bax | forward:GAACTGGGGGAGGATTGTGG | NM_009741.5 | 80 |
|  | reverse:GGGGTGACATCTCCCTGTTG |  |  |
| caspase-3 | forward:ACATGGGAGCAAGTCAGTGG | NM_001284409.1 | 149 |
|  | reverse:CGTCCACATCCGTACCAGAG |  |  |

**Supplementary Table S4.**

Response Surface Methodology Results.

| **Run** | **Independent variable** | | | **response value** |
| --- | --- | --- | --- | --- |
|  | A: Enzyme addition, (%) | B:(Time, h) | C:  (Temperature, ℃) | Polysaccharide content of *Allium mongolicum*, (%) |
| 1 | 15 | 2.5 | 60 | 54.55 |
| 2 | 20 | 3.5 | 60 | 61.03 |
| 3 | 15 | 3.5 | 65 | 51.95 |
| 4 | 20 | 3.5 | 60 | 60.02 |
| 5 | 20 | 2.5 | 55 | 58.83 |
| 6 | 25 | 3.5 | 55 | 59.91 |
| 7 | 25 | 2.5 | 60 | 60.59 |
| 8 | 20 | 3.5 | 60 | 62.04 |
| 9 | 25 | 3.5 | 65 | 64.17 |
| 10 | 20 | 2.5 | 65 | 58.94 |
| 11 | 15 | 3.5 | 55 | 51.27 |
| 12 | 20 | 4.5 | 65 | 61.37 |
| 13 | 20 | 4.5 | 55 | 59.12 |
| 14 | 20 | 3.5 | 60 | 60.94 |
| 15 | 25 | 4.5 | 60 | 65.17 |
| 16 | 20 | 3.5 | 60 | 61.51 |
| 17 | 15 | 4.5 | 60 | 54.61 |
